# Glucose-to-potassium ratio has a non-linear J-shaped association with acute kidney injury risk in traumatic brain injury

**DOI:** 10.1038/s41598-026-57082-4

**Published:** 2026-06-12

**Authors:** Hao Wang, Qing Dao, Xiaomei Li, Meng Shi, Meili Yang, Cui Gu, Jihua Wang

**Affiliations:** Department of Neurology, Yuxi People’s Hospital, Hongta District, Yuxi, 653100 Yunnan China

**Keywords:** Traumatic brain injury, Acute kidney injury, Glucose-to-potassium ratio, Non-linear relationship, Risk prediction model, Biomarkers, Medical research, Nephrology, Neurology, Risk factors

## Abstract

Acute kidney injury (AKI) is a severe complication of traumatic brain injury (TBI) associated with poor prognosis. The glucose-to-potassium ratio (GPR), an emerging marker of metabolic stress, may play a role in post-TBI AKI, but its precise relationship is unclear. This retrospective cohort study investigated the association between early-admission GPR and AKI in 2,388 TBI patients from the MIMIC-IV database. Using multivariable logistic regression and restricted cubic spline (RCS) models, we explored both linear and non-linear associations. Of the patients, 56.7% (1,355/2,388) developed AKI. While a simple linear association between GPR and AKI was not significant after adjusting for confounders (*P* = 0.20), we discovered a significant non-linear "J-shaped" relationship (P for non-linearity = 0.012). The risk of AKI increased steeply once GPR surpassed an inflection point of approximately 30. A random forest model incorporating multiple clinical variables demonstrated significantly superior predictive performance (AUC = 0.808) compared to traditional logistic regression (AUC = 0.785; DeLong test *P* = 0.025). These findings reveal that a high GPR (> 30) acts as an independent non-linear risk indicator for post-TBI AKI, Rather than a direct driver of injury, an elevated GPR represents a valuable early warning signal for metabolic decompensation, suggesting clinicians should closely monitor high-risk patients to facilitate early recognition of AKI.

**Supplementary Information:**

The online version contains supplementary material available at 10.1038/s41598-026-57082-4.

## Introduction

Traumatic brain injury (TBI) is a leading cause of death and long-term disability worldwide, affecting approximately 50 million people annually^1^. In the intensive care unit (ICU), TBI patients frequently develop multi-organ dysfunction due to their complex pathophysiology, with acute kidney injury (AKI) being one of the most common and severe complications, occurring at rates of 9.4%-19%^2^. The clinical consequences of post-TBI AKI are devastating; it not only causes a sharp increase in in-hospital mortality from 17.2% to 52.1% but also significantly prolongs ICU length of stay and duration of mechanical ventilation, and is associated with poorer long-term neurological recovery in survivors^3–5^.Therefore, establishing an effective and reliable early warning system to identify high-risk individuals for AKI among TBI patients is crucial for guiding clinical decision-making, optimizing resource allocation, and improving final outcomes.

In recent years, the role of metabolic dysregulation in organ injury among critically ill patients has garnered increasing attention. For TBI patients, this metabolic disturbance is particularly pronounced and unique. TBI directly disrupts the blood–brain barrier and triggers a potent neuroendocrine storm, leading to more severe stress-induced hyperglycemia compared to other types of trauma^6,7^. Concurrently, the massive release of catecholamines and cellular injury not only induce glycemic excursions but also lead to complex potassium fluctuations, including hypokalemia caused by a rapid intracellular shift of potassium ions^8,9^. Consequently, the glucose and potassium levels in TBI patients collectively provide a window into the severity of their unique pathophysiological stress.

The glucose-to-potassium ratio (GPR), as an integrated metric of glycemic and potassium status, has been shown to have predictive value in various critical illnesses, such as heart failure with preserved ejection fraction, sepsis, hepatic encephalopathy with acute-on-chronic liver failure, and acute stroke^10–13^. However, the role of GPR in the specific context of TBI, particularly its association with distant organ injury like AKI, remains largely unexplored, and the nature of this relationship (linear or non-linear) is unknown.

This study aimed to systematically investigate the association between early-admission GPR and the risk of post-TBI AKI using a large ICU database. We hypothesized that the relationship between GPR and AKI risk is not simply linear. Therefore, the primary objectives of this study were to: (1) evaluate the overall association between GPR and AKI; (2) explore the potential non-linear relationship between GPR and AKI risk after adjusting for key clinical confounders using a restricted cubic spline model; and (3) develop and evaluate a clinical model for predicting post-TBI AKI.

## Results

### Patient selection and baseline characteristics

**Fig. 1:**
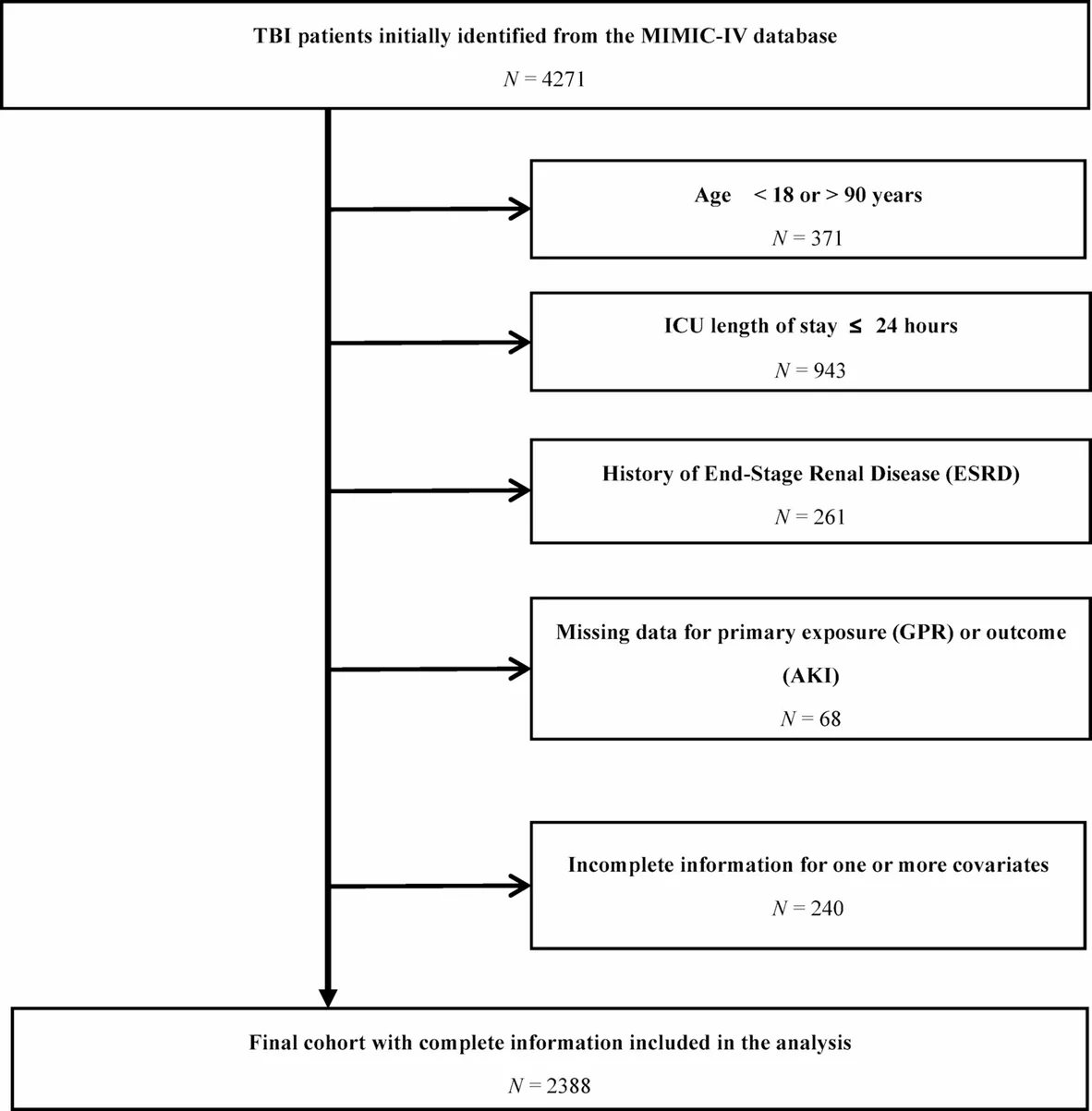
Flowchart of patient selection for the study. This flowchart illustrates the systematic screening and inclusion process of the traumatic brain injury (TBI) cohort from the MIMIC-IV database. After applying exclusion criteria (age, ICU stay duration, and history of renal disease), a final cohort of 2,388 patients was established.

The patient selection process for this study is detailed in Fig. 1 After an initial screening of 4,271 TBI patients, a final cohort of 2,388 patients with complete information was included in the analysis following the application of exclusion criteria. Among them, 1,355 (56.7%) patients developed any-stage AKI during their ICU stay.

As shown in Table 1, compared to patients without AKI, those who developed AKI were older (median age: 63 vs. 59 years), had a higher admission body weight (79.0 kg vs. 71.0 kg), and presented with a higher Charlson Comorbidity Index score (3 vs. 2) (all *P* < 0.001). Regarding specific comorbidities, congestive heart failure, cerebrovascular disease, liver disease, diabetes, and paraplegia were more prevalent in the AKI group (all *P* < 0.05). Furthermore, patients in the AKI group exhibited greater disease severity upon admission, as indicated by significantly higher APACHE-III, SOFA, and LODS scores, and were more frequently diagnosed with sepsis (57.5% vs. 29.1%) (all *P* < 0.001).

**Table 1:** Baseline characteristics of traumatic brain injury patients, stratified by the occurrence of acute kidney injury.

Characteristic	Overall (*N* = 2388)	No AKI (*N* = 1033)^1^	AKI (*N* = 1355)^1^	*P* value^2^
Glucose-to-potassium ratio (GPR)	31.25 [25.77, 38.90]	29.35 [24.74, 35.71]	32.86 [26.60, 41.47]	** < 0.001**
Age (years)	61.67 [42.97, 76.21]	59.12 [39.04, 74.30]	62.94 [45.55, 77.82]	** < 0.001**
Gender				**0.4**
Female	**810 (34%)**	**361 (35%)**	**449 (33%)**	
Male	**1578 (66%)**	**672 (65%)**	**906 (67%)**	
Race/Ethnicity				** < 0.001**
Asian	**56 (2.3%)**	**33 (3.2%)**	**23 (1.7%)**	
Black	**119 (5.0%)**	**57 (5.5%)**	**62 (4.6%)**	
Hispanic/Latino	**90 (3.8%)**	**49 (4.7%)**	**41 (3.0%)**	
Other/Unknown	**682 (29%)**	**253 (24%)**	**429 (32%)**	
White	**1441 (60%)**	**641 (62%)**	**800 (59%)**	
Admission weight (kg)	75.60[64.15, 88.00]	71.00[61.20, 83.00]	79.00[67.00, 92.40]	** < 0.001**
Charlson Comorbidity Index, Score	**3.00 [1.00, 4.00]**	**2.00 [0.00, 4.00]**	**3.00 [1.00, 5.00]**	** < 0.001**
Components^4^				
Myocardial infarction	**137 (5.7%)**	**50 (4.8%)**	**87 (6.4%)**	**0.10**
Congestive heart failure	**205 (8.6%)**	**59 (5.7%)**	**146 (11%)**	** < 0.001**
Peripheral vascular disease	**88 (3.7%)**	**30 (2.9%)**	**58 (4.3%)**	**0.077**
Cerebrovascular disease	**233 (9.8%)**	**82 (7.9%)**	**151 (11%)**	**0.009**
Dementia	**111 (4.6%)**	**45 (4.4%)**	**66 (4.9%)**	**0.6**
Chronic pulmonary disease	**250 (10%)**	**107 (10%)**	**143 (11%)**	**0.9**
Rheumatic disease	**37 (1.5%)**	**13 (1.3%)**	**24 (1.8%)**	**0.3**
Peptic ulcer disease	**19 (0.8%)**	**8 (0.8%)**	**11 (0.8%)**	** > 0.9**
Mild liver disease^5^	**155 (6.5%)**	**48 (4.6%)**	**107 (7.9%)**	**0.001**
Diabetes w/o complication^6^	**348 (15%)**	**123 (12%)**	**225 (17%)**	**0.001**
Diabetes w/ complication^6^	**57 (2.4%)**	**20 (1.9%)**	**37 (2.7%)**	**0.2**
Hemiplegia or paraplegia	**179 (7.5%)**	**58 (5.6%)**	**121 (8.9%)**	**0.002**
Malignant cancer	**81 (3.4%)**	**34 (3.3%)**	**47 (3.5%)**	**0.8**
Severe liver disease^7^	**47 (2.0%)**	**13 (1.3%)**	**34 (2.5%)**	**0.029**
Metastatic solid tumor	**28 (1.2%)**	**11 (1.1%)**	**17 (1.3%)**	**0.7**
Sepsis (Sepsis-3 criteria)^8^	**1,080 (45%)**	**301 (29%)**	**779 (57%)**	** < 0.001**
ICP monitoring	**111 (4.6%)**	**20 (1.9%)**	**91 (6.7%)**	** < 0.001**
Use of vasopressors	**85 (3.6%)**	**8 (0.8%)**	**77 (5.7%)**	** < 0.001**
Use of CRRT	**17 (0.7%)**	**0 (0%)**	**17 (1.3%)**	** < 0.001**
Use of RRT	**6 (0.3%)**	**0 (0%)**	**6 (0.4%)**	**0.040**
Use of invasive ventilation	**1,211 (51%)**	**320 (31%)**	**891 (66%)**	** < 0.001**
Use of mannitol	**207 (8.7%)**	**63 (6.1%)**	**144 (11%)**	** < 0.001**
Use of colloid bolus	**297 (12%)**	**49 (4.7%)**	**248 (18%)**	** < 0.001**
Use of ACEI/ARBs	**501 (21%)**	**206 (20%)**	**295 (22%)**	**0.3**
Use of vancomycin	**804 (34%)**	**204 (20%)**	**600 (44%)**	** < 0.001**
Use of antiplatelets	**377 (16%)**	**116 (11%)**	**261 (19%)**	** < 0.001**
Use of anticoagulants	**2,006 (84%)**	**833 (81%)**	**1,173 (87%)**	** < 0.001**
Use of statins	**587 (25%)**	**250 (24%)**	**337 (25%)**	**0.7**
First-day GCS, minimum	14.00 [13.00, 15.00]	14.00 [13.00, 15.00]	14.00 [12.00, 15.00]	**0.027**
First-day APACHE-III Score	34.00 [26.00, 44.00]	30.00 [24.00, 38.00]	37.00 [28.00, 50.00]	** < 0.001**
First-day SOFA Score	3.00 [2.00, 5.00]	2.00 [1.00, 4.00]	3.00 [2.00, 5.00]	** < 0.001**
First-day LODS Score	3.00 [2.00, 5.00]	2.00 [1.00, 3.00]	4.00 [2.00, 5.00]	** < 0.001**
First-day creatinine, max (mg/dL)^3^	0.90 [0.70, 1.10]	0.90 [0.70, 1.00]	0.90 [0.80, 1.20]	** < 0.001**
First-day BUN, max (mg/dL)	16.00 [12.00, 21.00]	14.00 [11.00, 19.00]	17.00 [12.00, 22.00]	** < 0.001**
First-day urine output (mL)	1,700.00 [1,150.0, 2,490.0]	1,890.00 [1,272.0, 2,675.0]	1,570.00 [1,085.0, 2,305.0]	** < 0.001**

AKI, acute kidney injury; GPR, glucose-to-potassium ratio; IQR, interquartile range; CCI, Charlson Comorbidity Index; RRT, renal replacement therapy; CRRT, continuous renal replacement therapy; GCS, Glasgow Coma Scale; APACHE, Acute Physiology and Chronic Health Evaluation; SOFA, Sequential Organ Failure Assessment; LODS, Logistic Organ Dysfunction System; BUN, blood urea nitrogen.

^1^Data are presented as Median [Q1, Q3] for continuous variables or as n (%) for categorical variables.

^2^*P* values were calculated using the Wilcoxon rank-sum test for continuous variables and the Chi-squared test or Fisher’s exact test for categorical variables.

^3^Identical median creatinine values (0.90 mg/dL) with a significant *P* value (*P* < 0.001) reflect the stochastic dominance of the AKI group’s distribution, particularly in the upper quartiles.

^4^Comorbidities were identified using ICD-9/10 codes algorithm^14^.

^5^Mild liver disease refers to chronic hepatitis or cirrhosis without portal hypertension.

^6^Diabetes without complications excludes end-organ damage, while diabetes with complications includes those with coded nephropathy, retinopathy, or neuropathy.

^7^Severe liver disease is defined by cirrhosis with portal hypertension accompanied by decompensated manifestations, such as esophageal varices, ascites, or hepatic encephalopathy.

^8^Sepsis-3 was defined according to the 2016 international consensus as suspected infection accompanied by an acute increase in the Sequential Organ Failure Assessment (SOFA) score of ≥ 2 points.

### Association between GPR and AKI

**Fig. 2:**
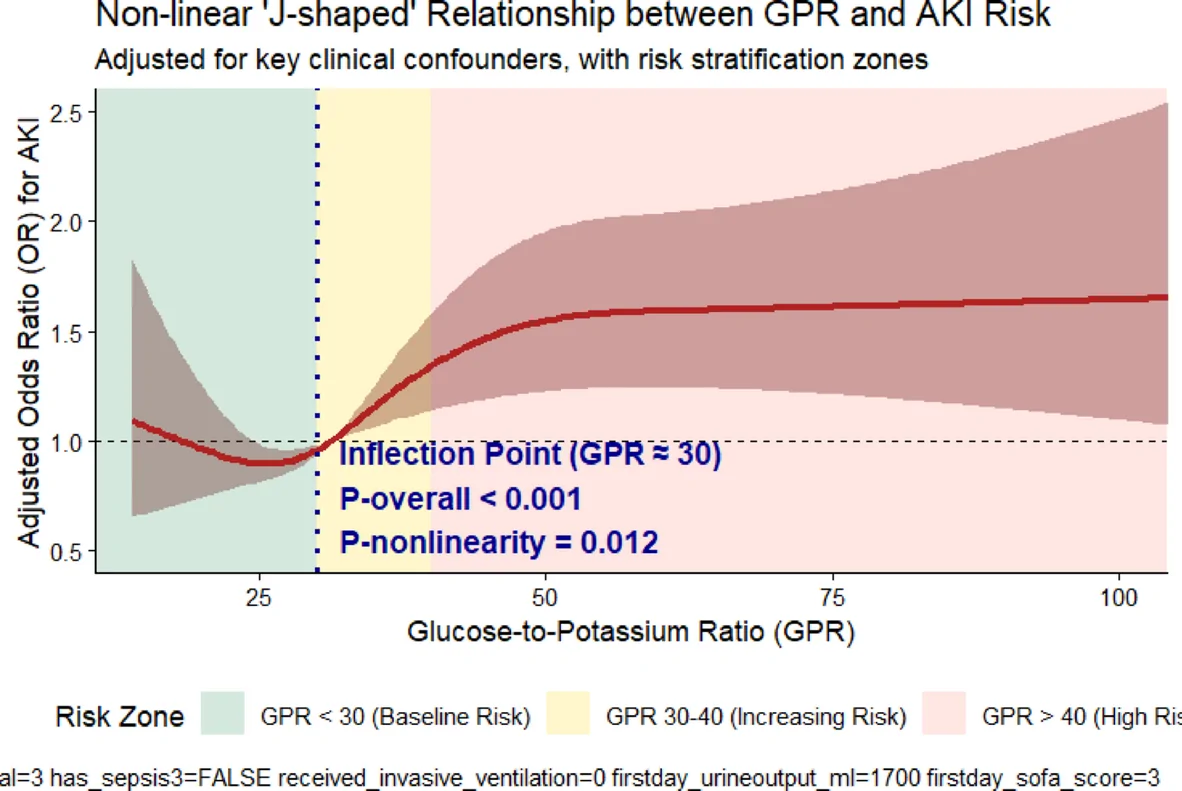
Non-linear "J-shaped" relationship between the Glucose-to-Potassium Ratio (GPR) and the risk of Acute Kidney Injury (AKI). Restricted cubic spline (RCS) plot adjusted for key clinical confounders (P_overall_ < 0.001, P_non-linearity_ = 0.012). The risk of AKI escalates sharply once the GPR surpasses the inflection point of approximately 30.

- *Univariable and multivariable linear regression analysis*: In the univariable analysis, the median GPR was significantly higher in the AKI group compared to the non-AKI group (33 vs. 29, *P* < 0.001). As detailed in Table 2, higher GPR values were significantly associated with an increased risk of AKI. However, in the multivariable logistic regression model that adjusted for all covariates, the linear association between GPR and AKI was not statistically significant (aOR per 1-SD increase = 1.09; 95% CI 0.96–1.25, *P* = 0.2). In contrast, admission weight, invasive mechanical ventilation, and APACHE-III score remained strong, independent predictors in this model.
- *Non-linear relationship exploration*: To further investigate the relationship pattern, we constructed a restricted cubic spline (RCS) model. The results indicated a significant overall association between GPR and AKI risk (*P* for overall association < 0.001), with a similarly significant non-linear component (*P* for non-linearity = 0.012). As depicted in Fig. 2, a clear "J-shaped" relationship emerged. The risk of AKI (adjusted OR) showed no significant change as GPR increased from low values to approximately 30; however, once GPR surpassed this inflection point, the risk began to rise steeply, reaching a high-risk plateau at a GPR value of around 60 (adjusted OR ≈ 1.6).

**Table 2:** Univariable and Multivariable Logistic Regression Analysis of Risk Factors for Any-stage AKI.

Characteristic	Univariable analysis			Multivariable analysis		
	OR	95% CI	*P* value	OR	95% CI	*P* value
GPR (per 1-SD increase)^1^	1.47	1.29, 1.68	< 0.001	1.09	0.96, 1.25	0.2
Age(per 1-SD increase)	1.01	1.00, 1.01	< 0.001	1.01	1.00, 1.03	0.13
Gender (Male vs. Female)^2^	1.08	0.91, 1.29	0.4	0.79	0.62, 1.00	0.046
Race/Ethnicity^2^						
Asian	–	–		–	–	
Black	1.56	0.82, 2.99	0.2	1.23	0.58, 2.64	0.6
Hispanic/Latino	1.20	0.61, 2.37	0.6	0.89	0.40, 1.98	0.8
Other/Unknown	2.43	1.40, 4.28	0.002	1.34	0.70, 2.62	0.4
White	1.79	1.05, 3.12	0.035	1.30	0.69, 2.49	0.4
Admission weight (per 1 kg increase)	1.02	1.02, 1.03	< 0.001	1.03	1.03, 1.04	< 0.001
Comorbidities						
Charlson Index (per 1-point)	1.10	1.07, 1.14	< 0.001	0.98	0.88, 1.09	0.6
Peripheral vascular disease	1.99	1.46, 2.75	< 0.001	1.47	0.98, 2.23	0.066
Cerebrovascular disease	1.50	0.96, 2.37	0.079	0.96	0.55, 1.67	0.9
Mild liver disease	1.45	1.10, 1.94	0.009	1.22	0.85, 1.76	0.3
Diabetes without complication	1.76	1.25, 2.52	0.002	1.21	0.73, 2.03	0.5
Paraplegia or hemiplegia	1.47	1.17, 1.87	0.001	1.11	0.80, 1.53	0.5
Severe liver disease	1.65	1.20, 2.29	0.003	1.27	0.82, 1.98	0.3
Clinical status & interventions						
Sepsis (Yes vs. No)^2^	3.29	2.77, 3.91	< 0.001	1.62	1.28, 2.04	< 0.001
ICP monitoring	3.65	2.28, 6.12	< 0.001	1.58	0.90, 2.89	0.13
Use of vasopressors	7.72	3.95, 17.4	< 0.001	2.50	1.07, 6.60	0.046
Invasive ventilation	4.28	3.60, 5.09	< 0.001	4.17	3.26, 5.35	< 0.001
Use of mannitol	1.83	1.35, 2.51	< 0.001	1.20	0.80, 1.80	0.4
Use of colloid bolus	4.50	3.30, 6.25	< 0.001	1.98	1.35, 2.96	< 0.001
Use of vancomycin	3.23	2.68, 3.90	< 0.001	1.37	1.07, 1.76	0.013
Use of antiplatelets	1.89	1.49, 2.39	< 0.001	1.18	0.89, 1.58	0.3
Use of anticoagulants	1.55	1.24, 1.93	< 0.001	1.08	0.82, 1.42	0.6
Severity & physiological markers						
GCS, min (per 1-point)	0.96	0.93, 0.99	0.006	1.06	1.01, 1.11	0.028
APACHE-III Score (per 1-point)	1.04	1.04, 1.05	< 0.001	1.03	1.02, 1.04	< 0.001
SOFA Score (per 1-point)	1.27	1.22, 1.32	< 0.001	0.99	0.93, 1.06	0.8
LODS Score (per 1-point)	1.40	1.34, 1.47	< 0.001	1.01	0.93, 1.09	0.8
Creatinine, max (per 1-mg/dL)	2.85	2.18, 3.76	< 0.001	0.95	0.69, 1.39	0.8
BUN, max (per 1-mg/dL)	1.04	1.03, 1.05	< 0.001	1.00	0.99, 1.02	0.6
Urine Output, 24 h (per 100-mL)^3^	1.00	1.00, 1.00	< 0.001	1.00	1.00, 1.00	< 0.001

OR, Odds Ratio; CI, Confidence Interval; GPR, Glucose-to-Potassium Ratio; SD, Standard Deviation; GCS, Glasgow Coma Scale; APACHE, Acute Physiology and Chronic Health Evaluation; SOFA, Sequential Organ Failure Assessment; LODS, Logistic Organ Dysfunction System; BUN, Blood Urea Nitrogen; cc, Complication.

^1^The OR for GPR is reported per 1-standard deviation increase. The multivariable analysis result for GPR is listed in the first row for prominence.

^2^For categorical variables, the reference group is the one not listed (e.g., for Gender, Female is the reference; for Sepsis, ‘No’ is the reference; for Race/Ethnicity, White is the reference). Multivariable results for Race/Ethnicity are omitted for brevity as the variable was not significant overall.

^3^The OR for Urine Output is reported per 100-mL increase. The ORs for other continuous variables are per 1-unit increase. The multivariable model was adjusted for all variables listed in this table.

### Sensitivity analysis

**Fig. 3:**
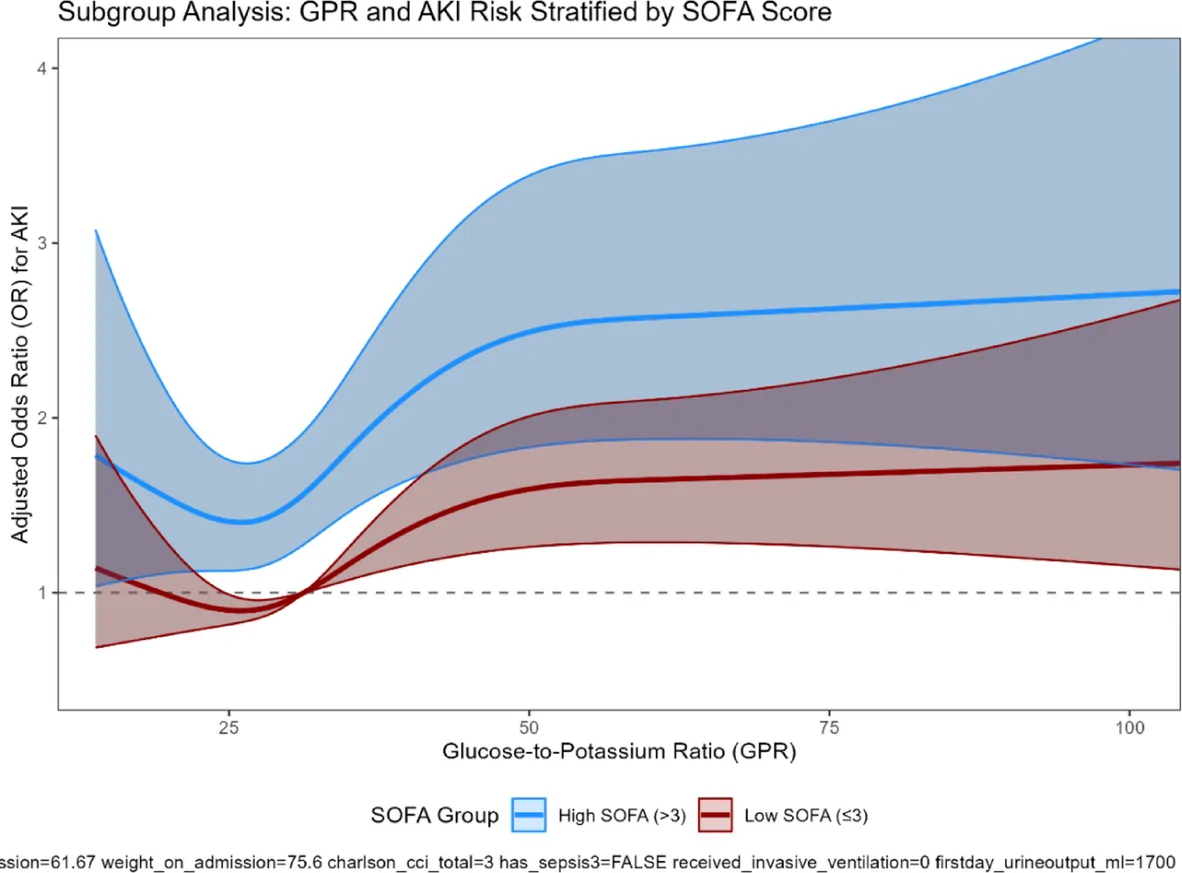
Subgroup analysis of the association between GPR and AKI risk, stratified by SOFA score. The non-linear "J-shaped" association between GPR and AKI risk remained consistent across different levels of disease severity (Low SOFA ≤ 3 vs. High SOFA > 3), with no significant interaction observed (P_interaction_ = 0.477).

We performed a sensitivity analysis stratified by the median SOFA score to test the stability of the GPR effect. As shown in Fig. 3, a similar "J-shaped" pattern was consistently observed in both the low and high SOFA score subgroups. Although the upward trend of risk appeared visually steeper in the high SOFA group, the test for interaction did not reach statistical significance (P for interaction = 0.477), suggesting that the "J-shaped" relationship is robust across different levels of disease severity. To further validate our findings, an additional sensitivity analysis was performed by incorporating administrative proxies for diuretic use and gastrointestinal losses into the multivariable model. The results confirmed that the independent association between GPR and AKI remained stable and statistically significant (Supplementary Table S3).

### Predictive model performance

**Fig. 4:**
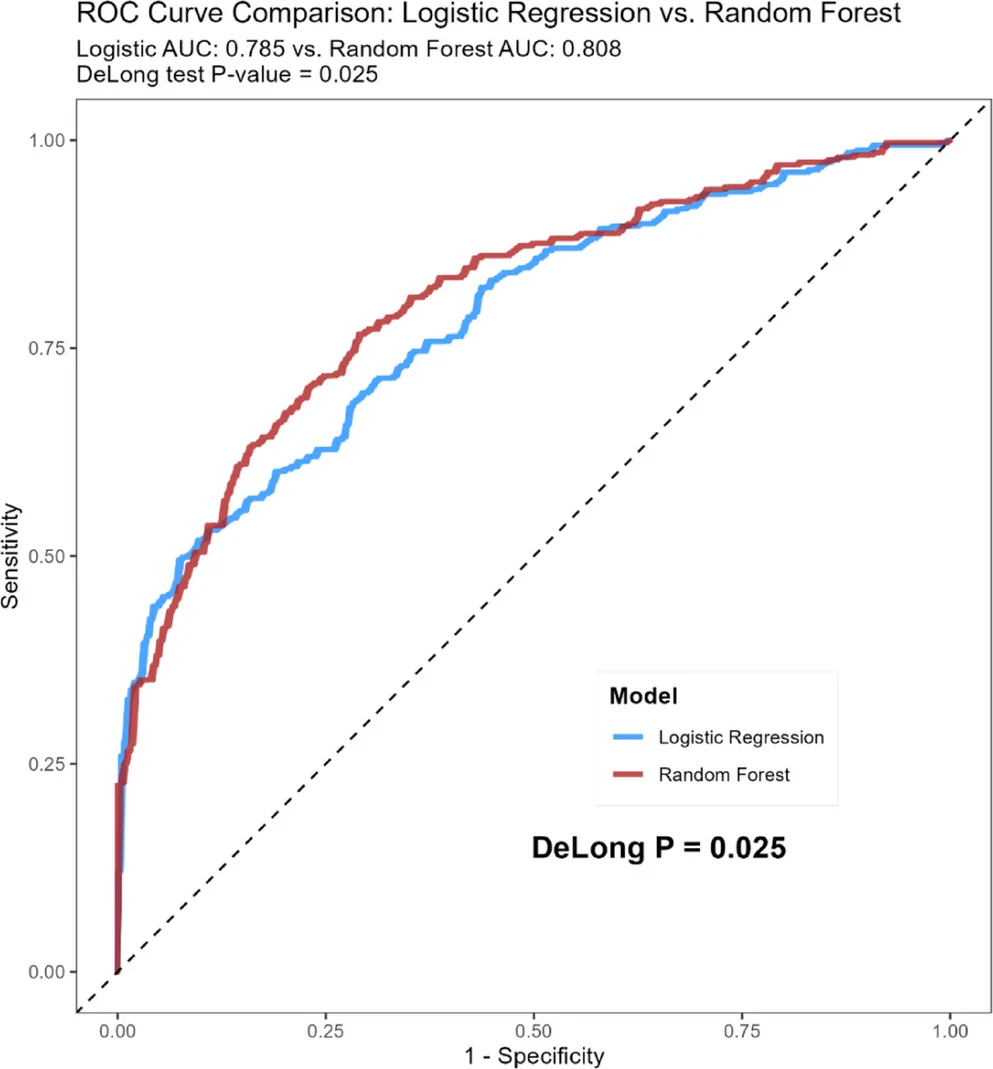
Performance comparison of the logistic regression and random forest models for predicting AKI. The random forest model achieved a significantly higher AUC (0.808) compared to the standard logistic regression model (0.785). The statistical superiority was confirmed by a formal DeLong test (*P* = 0.025).

**Fig. 5:**
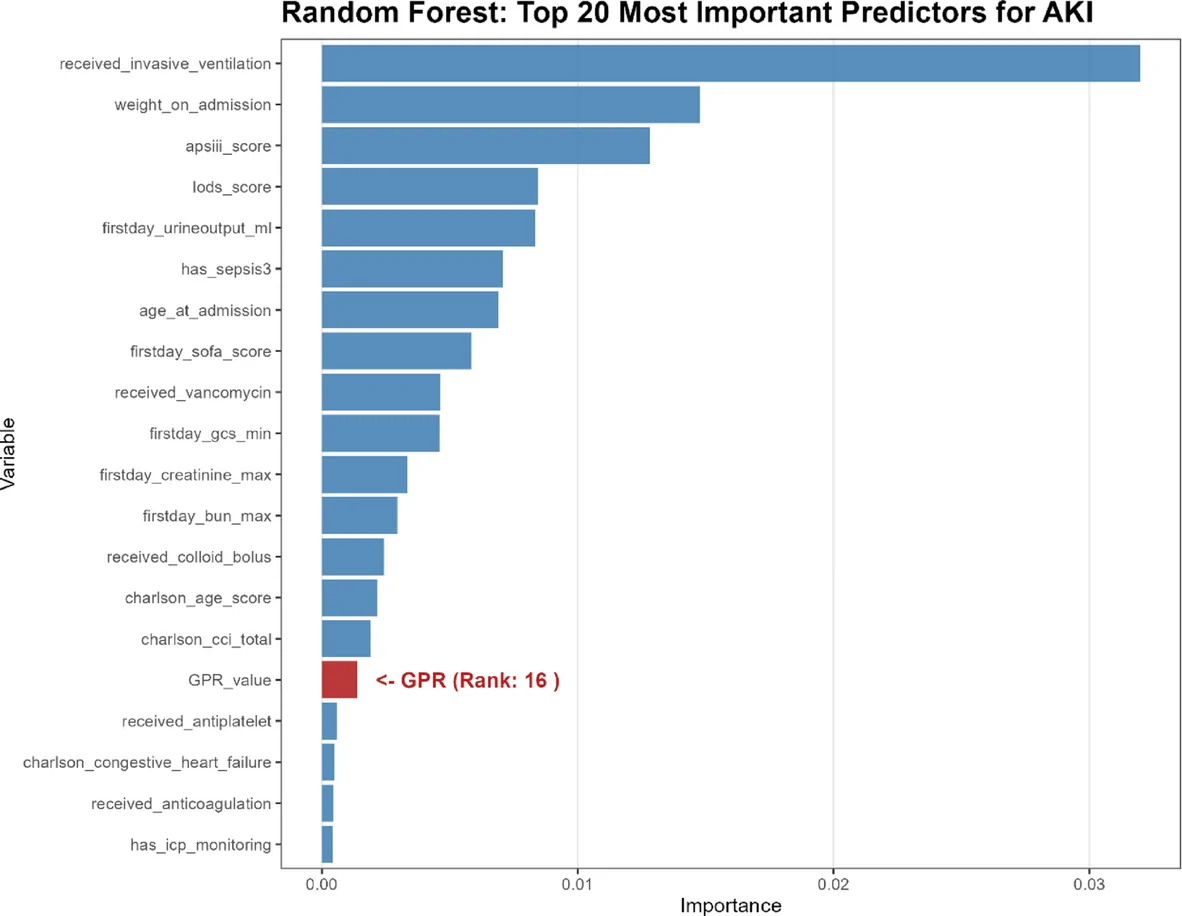
Top 20 most important predictors in the random forest model. Top 20 most important predictors identified via the permutation method. While treatment intensity (ventilation) and disease severity (APACHE-III) are dominant, the GPR (Rank 16) serves as a valuable adjunct metabolic signal.

The random forest model we developed demonstrated excellent predictive performance on the independent test set, achieving an AUC of 0.808. As illustrated in Fig. 4, its performance was significantly superior to that of the standard logistic regression model (AUC = 0.785), confirmed by a formal DeLong test (*P* = 0.025).The variable importance analysis (Fig. 5) revealed that invasive mechanical ventilation, admission weight, and APACHE-III score were the top three most important predictors while the importance of GPR was ranked relatively low (16th), which is consistent with our multivariable regression analysis.

### Model calibration and clinical utility

The calibration curve analysis indicated that our random forest model was well-calibrated, with its predicted probabilities closely aligning with the observed frequencies of AKI (see Supplementary Fig. S1). The Decision Curve Analysis (DCA) (Supplementary Fig. S2) demonstrated that using either the random forest or logistic regression model to guide clinical decisions provides a superior net benefit compared to the strategies of “treating all” or “treating none” across a wide range of risk thresholds (approximately 5% to 80%). Notably, the random forest model exhibited a slightly higher net benefit across most thresholds, confirming its excellent potential for clinical application.

## Discussion

This study is the first to systematically reveal a complex, non-linear association between the early-admission glucose-to-potassium ratio (GPR) and acute kidney injury (AKI) following TBI. In a large cohort of 2,388 TBI patients, we discovered that while GPR shows significant predictive value in univariable analysis, its true clinical significance is not that of an independent linear driver, but rather a threshold-based warning signal reflecting metabolic dysregulation. This finding holds significant potential value for the risk stratification and clinical monitoring of TBI patients.

First, our analysis clarifies the fundamental nature of GPR’s predictive value: it acts as a powerful confounding indicator rather than an independent risk factor. When strong confounders such as disease severity (e.g., SOFA score), treatment intensity (e.g., invasive ventilation), and complications (e.g., sepsis) were included in the model, the independent linear association of GPR was non-significant (aOR per 1-SD increase = 1.09; 95% CI 0.96–1.25; *P* = 0.2). This result strongly suggests that the apparent association between GPR and AKI is largely mediated by the patient’s overall critical condition^15^. In other words, GPR does not directly cause AKI but is highly correlated with the core pathophysiological processes that truly drive AKI, such as systemic inflammation, hypoperfusion, and organ failure^16–18^.

Second, the most significant finding of this study is the remarkable "J-shaped" dose–response relationship between GPR and AKI risk. The restricted cubic spline (RCS) analysis (P for non-linearity = 0.012) clearly delineated that the risk of AKI begins to escalate sharply once GPR surpasses an inflection point of approximately 30. This non-linear association remained robust after adjusting for all key covariates, and our sensitivity analysis confirmed that this "J-shaped" pattern is consistent across patients with different levels of disease severity^11,15,19,20^. This threshold effect has profound clinical and physiological implications. From a physiological perspective, a GPR > 30 may signify a critical tipping point of a "dual metabolic crisis": on one hand, stress-induced hyperglycemia can exacerbate tubular injury by inducing mitochondrial dysfunction; on the other, the accompanying relative potassium imbalance can impair renal concentrating capacity, with both factors synergistically amplifying the kidney’s susceptibility to ischemia–reperfusion injury^21,22^. From a clinical practice standpoint, the inflection point of GPR = 30 aligns closely with the critical decision-making thresholds for managing glucose (target upper limit 150–180 mg/dL) and potassium (warning lower limit 3.5 mmol/L) in the ICU^23–25^.Therefore, we propose that a GPR > 30 should be regarded as an integrative alarm for metabolic decompensation, whose primary clinical value lies in rapidly identifying high-risk patients who require immediate inclusion in enhanced monitoring and management protocols.

While GPR provides early metabolic insights, other systemic inflammatory and stress-related biomarkers have also demonstrated robust risk stratification capabilities for AKI. For instance, the neutrophil-to-lymphocyte ratio (NLR) serves as a reliable predictive and prognostic marker for AKI across diverse settings^26^. Similarly, the prognostic nutritional index (PNI) integrates nutritional and inflammatory status to identify individuals at high risk for renal damage^27^. In highly consumptive thrombo-inflammatory states, such as severe COVID-19, the neutrophil-to-platelet ratio (NPR) has proven effective in early AKI prediction^28^. Furthermore, integrating cellular indices like the platelet-to-lymphocyte ratio (PLR) with established clinical scores improves prediction in vulnerable populations^29^. Future algorithms could achieve optimal accuracy by synthesizing hyperacute metabolic GPR thresholds with these dynamic thrombo-inflammatory cellular ratios.

Furthermore, our predictive models clarify the positioning of GPR within the modern critical care assessment framework: it is a beneficial supplement, not a cornerstone. The random forest model developed in this study demonstrated excellent predictive performance (AUC = 0.808), and its variable importance ranking reaffirmed that organ dysfunction, treatment intensity, and physiological reserve are the dominant determinants of AKI risk. GPR’s importance ranked relatively low (16th), which is consistent with findings from previous research in other critical illness domains, where comprehensive scoring systems far outperform any single metabolic parameter in predicting multi-organ failure^23–25,30^.Importantly, the AUC improvement achieved by the machine learning model was statistically significant (DeLong test *P* = 0.025), confirming the added value of capturing non-linear feature interactions. this does not negate the value of GPR. Given its simplicity of calculation and lack of additional cost, GPR can serve as an effective adjunct to existing severity scoring systems, especially in resource-limited settings or scenarios requiring rapid, bedside screening^31,32^.

Moreover, a series of advanced assessments were conducted, which underscored the robustness and clinical utility of our proposed models. The sensitivity analysis found no significant statistical interaction between GPR’s effect and disease severity, supporting the generalizability of the "J-shaped" relationship. Furthermore, the calibration curve analysis confirmed our random forest model’s good calibration, while the decision curve analysis strongly demonstrated that using this model for risk stratification provides a superior net benefit compared to a "treat-all" or "treat-none" strategy.

Several inherent constraints of this study warrant consideration. Primarily, the retrospective nature of the design precludes the complete elimination of unmeasured confounders. Specifically, our reliance on a complete case analysis led to the exclusion of approximately 10% of the cohort, a factor that could introduce selection bias. Regarding metabolic phenotypes, our categorization of diabetes through administrative codes lacks the pharmacological depth—such as specific insulin requirements or baseline glycated hemoglobin levels—needed to definitively separate chronic insulin resistance from hyperacute, stress-induced dysglycemia. Consequently, this limitation may lead to misclassification of diabetes severity and potential residual confounding, as unmeasured baseline glycemic control could independently influence GPR. Methodologically, while we conducted sensitivity analyses to account for potential external drivers of hypokalemia (Supplementary Table S3), the available administrative markers function as proxies that lack precise volumetric granularity. Moreover, certain relevant endocrine disorders, such as primary hyperaldosteronism, were not identified in our specific TBI cohort, precluding their statistical adjustment due to zero variance. Our sensitivity analysis confirmed that the primary effect of GPR remained statistically significant (*P* = 0.007) despite these potential confounders. Specifically regarding baseline renal function, the inherent nature of emergency trauma admissions meant that historical outpatient creatinine records were frequently unavailable. Consequently, we utilized the minimum SCr value during the first 24 h of admission as a surrogate; while this is a recognized and conservative approach in critical care databases like MIMIC-IV, it may result in a potential underestimation of hyperacute or community-acquired AKI that initiated prior to the initial laboratory draw. Finally, this investigation was centered on single admission-window GPR values, whereas capturing dynamic metabolic trajectories over time might offer more nuanced prognostic insights^20^. Given known physiological variations across ethnic groups^33^, the generalizability of our proposed GPR threshold of 30 requires further validation in diverse international cohorts to ensure its global clinical utility.

## Conclusion

This study reveals that the relationship between early-admission GPR and the risk of post-TBI AKI exhibits a significant "J-shaped" non-linear pattern, with a risk inflection point at approximately 30. Although the independent predictive value of GPR is mediated by stronger clinical confounders, a GPR > 30 should be considered a practical, bedside warning signal for metabolic dysregulation, necessitating the initiation of early AKI alerts and enhanced monitoring.

## Methods

### Study design and data source

This study was a retrospective cohort study based on the Medical Information Mart for Intensive Care IV (MIMIC-IV, version 3.1) database. This database contains de-identified health-related data from 546,280 patients admitted to the Beth Israel Deaconess Medical Center in Boston, Massachusetts, between 2008 and 2022^34,35^.The project was approved by the Institutional Review Boards (IRBs) of the Massachusetts Institute of Technology (MIT) and Beth Israel Deaconess Medical Center. The requirement for informed consent was waived as the data were de-identified. Author Hao Wang (Record ID: 47312816) gained access to the database after completing the required training modules provided by the National Institutes of Health. The conduct and reporting of this study adhered strictly to the Strengthening the Reporting of Observational Studies in Epidemiology (STROBE) statement^36^.

### Patient population

We identified all adult patients diagnosed with TBI and admitted to the ICU using International Classification of Diseases, Ninth and Tenth Revision (ICD-9 and ICD-10) codes (ICD-9: 850xx-854xx; ICD-10: S060xxxx-S066xxxx). Prior validation studies have demonstrated that the use of the ICD-10 intracranial injury code (S06) yields a high overall positive predictive value (PPV) of 80.2%, which increases to 96.9% when listed as the principal diagnosis, confirming its high accuracy for identifying true TBI cases^37^. Inclusion criteria were: (1) age between 18 and 90 years; (2) a clear diagnosis of TBI; and 3) an ICU length of stay exceeding 24 h.

Exclusion criteria were: (1) a diagnosis of AKI or a history of end-stage renal disease (ESRD) upon ICU admission; (2) no recorded serum glucose and potassium levels within the first 24 h of admission; and (3) missing data for the primary outcome or exposure variables.

The detailed patient selection process is illustrated in Fig. 1.

### Variable definitions

*Exposure variable:* The primary exposure, the glucose-to-potassium ratio (GPR), was calculated based on the first measured serum glucose and potassium levels within 24 h of ICU admission. Serum glucose was measured in mg/dL, and serum potassium was measured in mmol/L. The formula is: GPR = Serum Glucose (mg/dL)/Serum Potassium (mmol/L).

*Outcome variable:* The primary outcome was the development of any-stage AKI during the ICU stay. The diagnosis strictly following the 2012 Kidney Disease Improving Global Outcomes (KDIGO) criteria^38^, incorporating both dynamic serum creatinine (SCr) fluctuations and hourly urine output to maximize diagnostic sensitivity. For the accurate classification of AKI, baseline SCr was derived as the minimum value recorded during the first 24 h of ICU admission. This hierarchical derivation rule was implemented due to the frequent absence of historical outpatient records in the emergency trauma setting; this conservative surrogate was chosen to avoid misclassification and minimize the risk of overestimating AKI incidence in patients with occult pre-existing chronic kidney disease.

*Covariates:* To adjust for potential confounders, we extracted baseline and early clinical variables across five domains: (1) demographics (age, gender, race, admission weight); (2) comorbidities, quantified by the Charlson Comorbidity Index (CCI) total score and its components^39^; (3) disease severity, assessed by SOFA, APACHE-III, LODS scores, and the lowest GCS within the first 24 h; (4) key laboratory and physiological markers (maximum creatinine, maximum BUN, and 24-h total urine output); and (5) clinical status and interventions (Sepsis-3 diagnosis, invasive ventilation, vasopressor use, etc.). A detailed list of variables is available in the supplementary material.

### Bias control

We implemented several measures to minimize bias. By setting strict inclusion and exclusion criteria, we aimed to reduce selection bias. We mitigated information bias by extracting systematically recorded clinical data directly from the database, rather than through manual chart review. Most importantly, we controlled for confounding bias by adjusting for a large number of potential confounders in our multivariable regression models and by performing stratified sensitivity analyses.

### Statistical analysis

All statistical analyses were performed using R software (version 4.5.0; R Foundation for Statistical Computing, Vienna, Austria), with a two-sided *P* value < 0.05 considered statistically significant.

Baseline characteristics were described for the study cohort. Continuous variables were presented as median and interquartile range [M (Q1, Q3)], and categorical variables as counts and percentages [n (%)]. Comparisons between the AKI and non-AKI groups were conducted using the Wilcoxon rank-sum test or the Chi-square/Fisher’s exact test, as appropriate. This study employed a complete case analysis strategy: after an initial screening to exclude variables with > 20% missingness, we included only patients with complete information for all remaining covariates (n = 2388) in the final analysis to ensure that all models were based on the same cohort.

To investigate the association between GPR and AKI, we first performed univariable logistic regression for preliminary screening of potential confounders. Subsequently, a final multivariable logistic regression model was built, which included all variables with a *P* value < 0.1 in the univariable analysis, with age and gender being forcibly included. To facilitate the interpretation of its effect size, GPR was standardized before model inclusion, and its result was reported as the adjusted odds ratio (aOR) and 95% confidence interval (CI) per 1-standard deviation (SD) increase.

To explore non-linear relationships, we fitted a multivariable model using a restricted cubic spline (RCS) with 4 knots for the standardized GPR and used an analysis of variance (ANOVA) to test the significance of the non-linear component. Additionally, we performed a sensitivity analysis stratified by the median SOFA score.

Finally, to assess overall predictive performance, we developed a random forest model tuned via tenfold cross-validation and evaluated its Area Under the ROC Curve (AUC) and other performance metrics on an independent test set. Model discrimination improvement over standard logistic regression was formally evaluated using the DeLong test, Net Reclassification Improvement (NRI), and Integrated Discrimination Improvement (IDI).To further validate the model’s performance, we conducted a calibration assessment and a Decision Curve Analysis (DCA) to quantify its net benefit across different risk thresholds.

## Supplementary Information

Below is the link to the electronic supplementary material.

Supplementary Material 1

## Data Availability

The data that support the findings of this study are publicly available in the Medical Information Mart for Intensive Care IV (MIMIC-IV) database (version 3.1), which can be accessed at https://physionet.org/content/mimiciv/3.1/. Access to the database requires completion of the CITI “Data or Specimens Only Research” training course.
