# Supplementary material for: Glucose-to-potassium ratio has a non-linear J-shaped association with acute kidney injury risk in traumatic brain injury
Source: Sci Rep. 2026 Jun 12;16:26829. doi: 10.1038/s41598-026-57082-4 (PMC13518952; doi:10.1038/s41598-026-57082-4)
Supplement: Supplementary file 1 [file 41598_2026_57082_MOESM1_ESM.docx]

## **Supplementary Material**

- **Supplementary Figure S1. Calibration plot for the random forest model.**
  *Assessment of the agreement between the predicted probabilities of AKI and the actually observed event rates across deciles. The model demonstrated excellent calibration.*

**Supplementary Figure S2. Decision curve analysis (DCA) for the prediction of AKI.**
*DCA demonstrates that using the random forest model for risk stratification provides a superior clinical net benefit compared to "treat-all" or "treat-none" strategies across a wide range of risk thresholds (5%–80%).*

Supplementary Figure S3. AKI incidence across different GPR strata.

*This bar chart shows a clear stepwise increase in the incidence of any-stage AKI as the GPR increases, particularly for values exceeding 30*

Supplementary Table S3. Sensitivity Analysis Adjusting for Potential Unmeasured Confounders

| Variable | Prevalence in TBI Cohort (%) | Adjusted Odds Ratio (95% CI)¹ | *P*-value |
| --- | --- | --- | --- |
| Glucose-to-Potassium Ratio (GPR)² | — | 1.01 (1.00–1.02) | **0.007** |
| Loop or Thiazide Diuretic Use | 34.3% | 1.11 (0.91–1.36) | 0.304 |
| Acute Gastrointestinal Loss³ | 46.1% | 1.08 (0.89–1.31) | 0.424 |
| Endocrine Conditions⁴ | 0.0% | (Not estimable) | — |

**Abbreviations:** TBI, traumatic brain injury; GPR, glucose-to-potassium ratio; OR, Odds Ratio; CI, Confidence Interval.

¹ *The multivariable logistic regression model was adjusted for age, admission weight, Charlson Comorbidity Index score, sepsis-3 diagnosis, invasive mechanical ventilation, 24-hour urine output, and first-day SOFA score.*

² *For this sensitivity model, GPR was treated as a continuous, unscaled variable to demonstrate the absolute risk increase per unit change.*

³ *Gastrointestinal loss was defined administratively by documentation of emesis or gastrointestinal tube drainage.*

⁴ *Specific endocrine conditions (including Cushing’s syndrome and primary hyperaldosteronism) were identified in 0 cases within this cohort, precluding their inclusion as covariates due to zero variance.*
